# Classifying Cognitive Profiles Using Machine Learning with Privileged Information in Mild Cognitive Impairment

**DOI:** 10.3389/fncom.2016.00117

**Published:** 2016-11-17

**Authors:** Hanin H. Alahmadi, Yuan Shen, Shereen Fouad, Caroline Di B. Luft, Peter Bentham, Zoe Kourtzi, Peter Tino

**Affiliations:** ^1^School of Computer Science, The University of BirminghamBirmingham, UK; ^2^School of Dentistry, The University of BirminghamBirmingham, UK; ^3^School of Biological and Chemical Sciences, Queen Mary University of LondonLondon, UK; ^4^School of Clinical and Experimental Medicine, The University of BirminghamBirmingham, UK; ^5^Department of Psychology, The University of CambridgeCambridge, UK

**Keywords:** discriminative feature extraction, supervised metric learning, learning with privileged information, learning vector quantization, linear discriminant analysis, fMRI graph feature

## Abstract

Early diagnosis of dementia is critical for assessing disease progression and potential treatment. State-or-the-art machine learning techniques have been increasingly employed to take on this diagnostic task. In this study, we employed Generalized Matrix Learning Vector Quantization (GMLVQ) classifiers to discriminate patients with Mild Cognitive Impairment (MCI) from healthy controls based on their cognitive skills. Further, we adopted a “Learning with privileged information” approach to combine cognitive and fMRI data for the classification task. The resulting classifier operates solely on the cognitive data while it incorporates the fMRI data as privileged information (PI) during training. This novel classifier is of practical use as the collection of brain imaging data is not always possible with patients and older participants. MCI patients and healthy age-matched controls were trained to extract structure from temporal sequences. We ask whether machine learning classifiers can be used to discriminate patients from controls and whether differences between these groups relate to individual cognitive profiles. To this end, we tested participants in four cognitive tasks: working memory, cognitive inhibition, divided attention, and selective attention. We also collected fMRI data before and after training on a probabilistic sequence learning task and extracted fMRI responses and connectivity as features for machine learning classifiers. Our results show that the PI guided GMLVQ classifiers outperform the baseline classifier that only used the cognitive data. In addition, we found that for the baseline classifier, divided attention is the only relevant cognitive feature. When PI was incorporated, divided attention remained the most relevant feature while cognitive inhibition became also relevant for the task. Interestingly, this analysis for the fMRI GMLVQ classifier suggests that (1) when overall fMRI signal is used as inputs to the classifier, the post-training session is most relevant; and (2) when the graph feature reflecting underlying spatiotemporal fMRI pattern is used, the pre-training session is most relevant. Taken together these results suggest that brain connectivity before training and overall fMRI signal after training are both diagnostic of cognitive skills in MCI.

## 1. Introduction

Alzheimer's Disease (AD) is the most common neurodegenerative disease in ageing. It is characterized by the progressive impairment of neurons and their connections. Mild Cognitive Impairment (MCI) is the prodromal stage of AD. Thus, accurate diagnosis of MCI (i.e., the early stage of AD) is very important for timely treatment and delay of disease progression. As MCI results in detectable loss of cognitive function, cognitive test scores have been used diagnostically (Albert et al., 2010). Further, MCI is known to cause changes in brain activation patterns as well as in brain connectivity. Therefore, fMRI has been increasingly used as a diagnostic tool of MCI patients (Challis et al., 2015; Chen et al., 2015). In machine learning terms, diagnosis of MCI patients can be formulated as a classification task to discriminate MCI patients from healthy controls. In this paper, we present a novel classifier using cognitive test scores as inputs to the classifier and using fMRI data as privileged information.

In the recent literature on the classification tasks related to AD, we observe a clear trend: state-of-the-art machine learning techniques have been increasingly employed to take on new tasks. For example, a classification task should also provide insights into the relevance of the input features used for the task. In Challis et al. (2015), Gaussian process classifiers have been employed for the discrimination between healthy controls and MCI patients as well as the discrimination between MCI and AD patients. More importantly, Gaussian process classifiers have been used to automatically determine the relevant input features when training the classifier. In Chen et al. (2015), a challenging classification task was tested, that is, discrimination of two subgroups of MCI patients. Patients in one subgroup will likely progress to AD but those in another group will not convert to AD. In the literature, this classification task is referred to as MCI-AD conversion prediction. This work incorporates data from both healthy subjects and AD patients for classification of MCI patients using the transfer learning framework. Transfer learning is a (relatively) new development in machine learning that aims to boost the performance of a classifier operating in one domain (e.g., MCI patients) by incorporating data from other domains (e.g., healthy subjects and AD patients).

Here we ask whether MCI patients differ in their cognitive skills from controls. Our task is to classify cognitive profiles in patients vs. controls based on cognitive scores and fMRI data. Furthermore, we address the case when fMRI data are not available for classifying a new subject. To utilize the fMRI data for the task, we train our classifier on participants for whom both cognitive and fMRI data are available. After that, the trained classifier will classify a new subject solely based on his/er cognitive test scores. This case is of relevance in practice because (1) When compared to cognitive data, the collection of neuroimaging data is much more time-consuming and expensive; (2) Many older individuals (e.g., those with a cardiac pacemaker) may not be safe for imaging such as fMRI scanning. On the other hand, neuroimaging data have more diagnostic power than cognitive data and thus should be used when available. In our work, the classifier is trained by adopting a “metric learning” based approach to *Learning with Privileged Information* (LPI) (Fouad, 2013). As transfer learning, LPI is also a new development in machine learning. In our context, cognitive data are the inputs to the classifier. In contrast, fMRI data act as privileged information that is used only for training the classifier (along with the cognitive data). As most classifiers operate based on a distance/similarity measure between pairs of input vectors, the metric tensor used to compute such distance is therefore crucial for the classification task. In the model of Fouad (2013), the privileged information (in our case fMRI data) is used to modify the metric tensor (and hence the metric) in the original space (in our case cognitive test scores) to improve the classification accuracy in the original space. Intuitively, if cognitive test scores of two participants appear “similar," but their fMRI data shows different characteristics, the distance between the two cognitive test score vectors should be increased (and vice-versa). As the scale parameter in Challis et al. (2015), the diagonal elements of the discriminative metric tensor can be used to automatically determine the relevant cognitive features.

## 2. Materials

The cognitive and fMRI data used in this study were collected in the context of two behavioral and fMRI studies (Baker et al., 2015; Luft et al., 2015, 2016) in which the participants were asked to predict the orientation of a test stimulus following exposure to structured sequence of leftwards and rightwards oriented gratings, and no feedback were given. Both studies aimed to (1) test whether training on structured temporal sequences improves the ability to predict upcoming sensory events and (2) identify brain regions that support the ability of using implicit knowledge about the past for predicting future. In particular, Baker et al. (2015) and Luft et al. (2015) investigated how MCI patients differ from healthy controls in terms of (1) their ability to learn predictive structures as well as (2) their learning-dependent brain activation patterns. The diagnosis of MCI patients was made by an experienced consultant psychiatrist (PB) using the National Institute of Ageing and Alzheimer's association working group criteria (Albert et al., 2010).

In both studies, participants took part in two fMRI scans before and after behavioral training (i.e., pre- and post-training session) during which they completed 5–8 independent runs of the prediction task in each scanning session. Each run comprised 5 blocks of structured and 5 blocks of random sequences (3 trials per block) presented in a random counterbalanced order. In each trial, the participant was presented with a sequence of eight left and rightward oriented gratings (in rapid succession, 250 ms + fixation 200 ms) followed by a repeat of the same sequence. The participant was instructed to pay attention to the sequence and respond whether the test grating (randomly chosen grating during the second repeat) was correct or incorrect given that presented sequence. Even though the participants could not tell what exactly was the sequence structure, they learn how to correctly predict whether the grating has the correct orientation given the presented sequence. In random sequence trials, the grating's orientations were randomly generated so the participant could not correctly predict them.

The fMRI data used in this study were acquired in a 3T Achieva Philips scanner at the Birmingham University Imaging Center using a 30 two-channel head coil. Anatomical images were obtained using a sagittal three dimensional T1-weighted sequence with 175 slices (voxel size = 1 × 1 × 1 mm^3^) for localization and visualization of functional data. Functional data were acquired using a T2-weighted EPI sequence with 32 slices (whole-brain coverage; TR = 2 s; TE = 35 ms; flip angle = 73; voxel size = 2.5 × 2.5 × 4 mm^3^).

In Luft et al. (2016), regions-of-interest (ROIs) were identified by applying whole-brain general linear model analysis with a voxel-wise mixed-design three-way ANOVA, that is,

$$\begin{array}{l} \text{session }(\text{pre- vs}.\text{ post-training})\times \text{sequence }(\text{structured vs}.\\ \text{ random})\times \text{group }(\text{MCI vs}.\text{ controls}). \end{array}$$

Statistical maps were cluster threshold corrected (*p* < 0.05). Table 1 in Luft et al. (2016) listed all brain regions showing significant interaction between session, sequence, and group. For the study presented in this paper, we combined two ROIs in the frontal region (Superior Frontal Gyrus, SFG, on the right hemisphere and Medial Frontal Gyrus, MFG, on the left hemisphere) and two ROIs in the cerebellar region (Cerebellar Lingual and Cullmen ROIs in both hemispheres). This resulted in a frontal ROI of size 126 and a cerebellar ROI of size 82. Also, a subcortical ROI (that is, the parahippocampal gyrus ROI of size 32) was selected for the study.

All 60 participants involved in this study had undergone cognitive skill tests (including working memory, cognitive inhibition and attentional skills). These tests provide four quantitative measures of different cognitive skills for each participant:

1. In the working memory task, a number of colored dots are on display for half second. Then, they disappear for 1 s and reappear with some dots having changed their color. A participant is asked to judge whether a given dot has changed its color or not. The participant's working memory skill can be measured by the maximal number of colored dots on display for achieving a 70.7% test performance (denoted by *n*_dots_);
2. To quantify a participant's attention skill, the following cognitive task was performed: two objects are on display, one located at the display center, another located on the periphery of the display. The peripheral object can only take one of eight equally distributed radial directions (with respect to the display center). The central object could be either car or truck silhouette, whereas the peripheral object must always be the truck silhouette. The participant was asked to identify the type of the central object (car vs. truck) and the location of the peripheral stimulus before the display was masked by white visual noise. This skill is measured by the minimal display time required for the participant to achieve 70% task performance. Depending on whether or not there are distractors on the display, the skill of divided or selective attention is measured (denoted by $t_{\text{disp}}^{d}$ and $t_{\text{disp}}^{s}$, respectively);
3. The skill of inhibition is measured in a stop-signal test. A participant is first cued to perform a motor task. This is followed by a tone with some time delay, which signals task abortion. The quantity measuring the inhibition skill, *t*_delay_, is given by the minimum delay time for achieving a 70.7% test performance.

Sixty participants are involved in this study. Thirty-four of them have both cognitive and fMRI data. Among these participants, nine MCI patients and nine healthy controls come from the cohort reported in Luft et al. (2015). The remaining 16 healthy controls come from the cohort reported in Luft et al. (2016). The size of that cohort is 20. Four of them are not included in this study because their cognitive data were missing. Note that for these 34 subjects having both cognitive and neuroimaging data for training of classifiers, MCI patients and healthy controls were age matched: mean age of MCI patients was 68.9, and mean age of controls was 68.3. The remaining 26 participants have cognitive data only. Among them, four MCI patients and five healthy controls come from Baker et al. (2015) and Luft et al. (2015). The remaining 17 participants are from unpublished studies but they participated exactly the same experiments as other participants. Note that all neuroimaging data used in this study are reported either in Luft et al. (2015) or in Luft et al. (2016).

## 3. Methods

### 3.1. Generation of fMRI features

#### 3.1.1. fMRI signal features

For each ROI and each (pre- and post-training) session, we calculated percent signal change (PSC) by subtracting fMRI responses to random sequences from fMRI response to structured sequences and dividing by averaged fMRI response to both stimulus sequences. Let *n*_*r*_ and *n*_*s*_ denote the number of volumes scanned during the trials with random and structured sequences, respectively. For a ROI of size *V*, its PSC value is computed as follows:

(1) $$\begin{array}{l} PSC=\frac{1}{V}\overset{V}{\underset{v=1}{\sum }}\frac{\frac{1}{n_{s}}\underset{i\in I_{s}}{\sum }y_{vi}-\frac{1}{n_{r}}\underset{j\in I_{r}}{\sum }y_{vj}}{\frac{1}{n_{s}}\underset{i\in I_{s}}{\sum }y_{vi}+\frac{1}{n_{r}}\underset{j\in I_{r}}{\sum }y_{vj}} \end{array}$$

where *i* and *j* denote volume index, *v* voxel index, *I*_*s*_ = {*i*_1_, …, *i*_*n*_*s*__} the collection of “structured” volumes and *I*_*r*_ = {*j*_1_, …, *j*_*n*_*s*__} the collection of “random” volumes. The above definition implies that PSC measures scaled fMRI-response to temporally structured stimuli and it is an overall measure averaged over both volumes and voxels.

#### 3.1.2. fMRI graph features

##### 3.1.2.1. Graph matrix

Graph structure characterizes the connectivity between nodes of a graph. In this study, the graph structure of a single ROI is represented by so-called graph matrix *G* of size *V* × *V* where *V* denotes the ROI size. The value of *G*_*ij*_ measures the functional connectivity between voxel *i* and voxel *j*, and is computed as (linear) cross-correlation between two fMRI time series of length *n* on the voxel pair (denoted by ${\text{y}}_{i}={\left(y_{i1},\ldots ,y_{in}\right)}^{⊺}$ and ${\text{y}}_{j}={\left(y_{j1},\ldots ,y_{jn}\right)}^{⊺}$, respectively), that is,

(2) $$\begin{array}{l} G_{ij}=\frac{1}{n}\cdot \frac{\overset{n}{\underset{k=1}{\sum }}\left(y_{ik}-\mu_{i}\right)\cdot \left(y_{jk}-\mu_{j}\right)}{\sigma_{i}\cdot \sigma_{j}} \end{array}$$

where μ and σ stand for the mean and standard deviation of individual fMRI time series. In the case of *i* = *j*, we obtain *G*_*ij*_ = 1. Note that *G*_*ij*_ is a connectivity measure independent of the activation intensity on each of two voxels.

##### 3.1.2.2. Discriminative feature extraction

Often, a classifier's inputs are not those raw data to be classified but the features extracted from the raw data. This can significantly reduce the input dimension, which tackles both “curse of dimensionality” and the small sample-size problem. Therefore, a good choice of feature vector plays an important role in classification. This is the motivation for extraction of discriminative features. The discriminative features are suitable because they are extracted in a task-driven and supervised manner. Linear Discriminant Analysis (LDA) is a machine learning technique for discriminative feature extraction. The assumption of LDA is that the feature vectors of each class are Gaussian-distributed. In LDA, high-dimensional feature vectors are projected into a lower-dimensional space and the projection matrix is optimized so that the classes are maximally separated in the projection space. To this end, the empirical covariance matrices need to be estimated using the feature vectors from individual classes. If the number of feature vectors is small and their dimension is high, the empirical estimates of covariance matrices are not accurate. Thus, LDA suffers from the same problem as classifiers do. So-called 2D-LDA has been proposed by Sato et al. (2008) for the cases where data items are matrices (e.g., graph matrices in this study) and a direct application of standard LDA with vectorized matrices could fail due to the above-mentioned problem. In the following, we summarize both standard LDA and 2D-LDA with the dimension of the projection space fixed to one.

For standard LDA, assume that we have *N d*-dimensional feature vectors, {**x**_*n*_ : *n* = 1, …, *N*}, for training in which *N*_1_ feature vectors are from *Class 1* and *N*_2_ = *N* − *N*_1_ from *Class 2*. Denote these two subsets by 𝒞_1_ and 𝒞_2_, respectively. The mean vectors of *Class 1* and *Class 2* are given by $m_{1}=\frac{1}{N_{1}}\sum_{X_{n}\in {𝒞}_{1}}X_{n}$ and $m_{2}=\frac{1}{N_{2}}\sum_{X_{n}\in {𝒞}_{2}}X_{n}$, respectively. Define the between-class covariance matrix **S**_*B*_ and the total within-class covariance matrix **S**_*W*_ as

(3) $$\begin{array}{l} {\text{S}}_{B}=\left({\text{m}}_{2}-{\text{m}}_{1}\right){\left({\text{m}}_{2}-{\text{m}}_{1}\right)}^{⊺} \end{array}$$

and

(4) $$\begin{array}{l} {\text{S}}_{W}=\underset{{\text{x}}_{n}\in {𝒞}_{1}}{\sum }\left({\text{x}}_{n}-{\text{m}}_{1}\right){\left({\text{x}}_{n}-{\text{m}}_{1}\right)}^{⊺}+\underset{{\text{x}}_{n}\in {𝒞}_{2}}{\sum }\left({\text{x}}_{n}-{\text{m}}_{2}\right){\left({\text{x}}_{n}-{\text{m}}_{2}\right)}^{⊺}. \end{array}$$

The projection matrix **w** of size *d* × 1 is optimized by maximizing the Fisher criterion defined by

(5) $$\begin{array}{l} J\left(\text{w}\right)=\frac{{\text{w}}^{⊺}{\text{S}}_{B}\text{w}}{{\text{w}}^{⊺}{\text{S}}_{W}\text{w}}=\frac{{\text{D}}_{B}}{{\text{D}}_{W}}. \end{array}$$

**D**_*B*_ and **D**_*W*_ are referred to as the between-class distance and the total within-class distance. Denote the optimized **w** by **w**_opt_ and the extracted features are given as $\left\{f_{n}={\text{w}}_{\text{opt}}^{⊺}{\text{x}}_{n}:n=1,\ldots ,N\right\}$.

For 2D-LDA, assume that we have *N* graph matrices of size *d* × *d*, {**X**_*n*_ : *n* = 1, …, *N*}, for training in which *N*_1_ feature vectors are from *Class 1* and *N*_2_ = *N* − *N*_1_ from *Class 2*. Denote these two subsets by 𝒞_1_ and 𝒞_2_, respectively. For *Class 1* and *Class 2*, their mean matrices are given by $M_{1}=\frac{1}{N_{1}}\sum_{X_{n}\in {𝒞}_{1}}X_{n}$ and $M_{2}=\frac{1}{N_{2}}\sum_{X_{n}\in {𝒞}_{2}}X_{n}$. In contrast to standard LDA, we need two (left and right) projection matrices (or vectors), denoted by **a** and **b** of size *d* × 1 projecting the matrices into real numbers. Similarly, the between-class distance and the total within-class distance are defined as

(6) $$\begin{array}{l} {\text{D}}_{B}={\text{a}}^{⊺}\left({\text{M}}_{2}-{\text{M}}_{1}\right)\text{b}{\text{b}}^{⊺}\left({\text{M}}_{2}-{\text{M}}_{1}\right)\text{a} \end{array}$$

(7) $$\begin{array}{l} ={\text{b}}^{⊺}\left({\text{M}}_{2}-{\text{M}}_{1}\right)\text{a}{\text{a}}^{⊺}\left({\text{M}}_{2}-{\text{M}}_{1}\right)\text{b} \end{array}$$

and

(8) $$\begin{array}{l} {\text{D}}_{W}=\underset{{\text{X}}_{n}\in {𝒞}_{1}}{\sum }{\text{a}}^{⊺}\left({\text{X}}_{n}-{\text{M}}_{1}\right)\text{b}{\text{b}}^{⊺}\left({\text{X}}_{n}-{\text{M}}_{1}\right)\text{a}\\ +\underset{{\text{X}}_{n}\in {𝒞}_{2}}{\sum }{\text{a}}^{⊺}\left({\text{X}}_{n}-{\text{M}}_{2}\right)\text{b}{\text{b}}^{⊺}\left({\text{X}}_{n}-{\text{M}}_{2}\right)\text{a} \end{array}$$

(9) $$\begin{array}{l} =\underset{{\text{X}}_{n}\in {𝒞}_{1}}{\sum }{\text{b}}^{⊺}\left({\text{X}}_{n}-{\text{M}}_{1}\right)\text{a}{\text{a}}^{⊺}\left({\text{X}}_{n}-{\text{M}}_{1}\right)\text{b}\\ +\underset{{\text{X}}_{n}\in {𝒞}_{2}}{\sum }\text{b}\left({\text{X}}_{n}-{\text{M}}_{2}\right)\text{a}{\text{a}}^{⊺}\left({\text{X}}_{n}-{\text{M}}_{2}\right)\text{b}. \end{array}$$

Note that **M**_1_, **M**_2_, and **X**_*n*_, *n* = 1, 2, …, *N*, are all symmetric matrix. The projection vectors **a** and **b** are optimized by maximizing *J*(**a**, **b**) = **D**_*B*_/**D**_*W*_ iteratively. At each iteration, we optimize **a** or **b** while keeping **b** or **a** fixed. This procedure is repeated until *J* has converged. Denote the optimized **a** and **b** by **a**_opt_ and **b**_opt_. The extracted features are given as $\left\{f_{n}={\text{a}}_{\text{opt}}^{⊺}{\text{X}}_{n}{\text{b}}_{\text{opt}}:n=1,\ldots ,N\right\}$.

Note that the number of free parameters to be optimized is *d*^2^ for standard LDA operating on vectorized graph matrices and 2*d* for 2D-LDA operating on graph matrices directly.

##### 3.1.2.3. Small sample-size problem

The main idea of this study is using costly but informative fMRI measurements as valuable privileged information in a classification task operating on cognitive features only. To do so the complex spatial-temporal structure in fMRI signals will need to be transformed into a set of indexes (scalars) that best discriminate between the classes.

In our approach we first capture the spatial-temporal structure of fMRI signals within an ROI as a cross-correlation graph. An ROI of *V* voxels will be represented as a full undirected graph with *n* nodes (one for each voxel) and the edge between nodes *i* and *j* is weighted by the value of the correlation coefficient between fMRI signals in the two voxels. Each such graph will in turn be represented by an *V* × *V* symmetric matrix **X** collecting the edge weights.

In this study we have two classes of *N* subjects - *N*_*p*_ patients and *N*_*c*_ healthy controls (that is *N* = *N*_*p*_ + *N*_*c*_). The graph matrices of patients and controls are collected in matrix sets 𝒞_*p*_ and 𝒞_*c*_. Given the two sets of matrices, we propose to extract the discriminating feature *f* through a quadratic form applied to graph matrix **X**: *f* = **a**^⊺^**Xb**. Both **a** and **b** are a *V*-dimensional vectors determined via an optimization problem expressing the need to maximally separate the two classes, while keeping the within-class variability minimal. To find the projection vectors **a** and **b** we used 2D-LDA (Ye et al., 2004).

For an ROI with *V* voxels, the discriminative features **a** and **b** are *V*-dimensional vectors, meaning that when determining **a** and **b** we have 2*V* free parameters. As the number of subjects *N* is smaller than 2*V*, in order to avoid overfitting, the size of the graph representing spatial-temporal structure of cortical activations in that ROI needs to be reduced. Note that in our original formulation, each element *a*_*i*_ of **a** corresponds to a particular voxel *i* whose spatial position is **r**_*i*_. It is natural to expect that spatially close voxels will have similar activation patterns. We therefore introduce a set of *K* spatially smoothing Gaussian kernels $N\left(\text{r};\mu_{k},\Sigma_{k}\right)$, *k* = 1, 2, …, *K*, in the voxel space, positioned at **μ**_*k*_, shape determined by the covariance matrix **Σ**_*k*_. This leads to a decomposition:

(10) $$\begin{array}{l} a_{i}=\overset{K}{\underset{k=1}{\sum }}{ã}_{k}N\left({\text{r}}_{i};\mu_{k},\Sigma_{k}\right) \end{array}$$

The values of the smoothing kernels *k* at each voxel *i* can be collected in the smoothing matrix.

(11) $$\begin{array}{l} {\text{P}}_{i,k}=N\left({\text{r}}_{i};\mu_{k},\Sigma_{k}\right) \end{array}$$

The feature vectors **a** and **b** can then be written as $\text{a}=\text{P}\tilde{\text{a}}$ and $\text{b}=\text{P}\tilde{\text{b}}$, respectively. We have:

(12) $$\begin{array}{l} f={\text{a}}^{⊺}\text{X}\text{b}={\tilde{\text{a}}}^{⊺}{\text{P}}^{⊺}\text{X}\text{P}\tilde{\text{b}} \end{array}$$

The *V* × *V* graph matrix **X** is thus reduced to the *K* × *K* matrix

(13) $$\begin{array}{l} \tilde{\text{X}}=\text{P}\text{X}{\text{P}}^{⊺} \end{array}$$

and

(14) $$\begin{array}{l} f={\tilde{\text{a}}}^{⊺}\tilde{\text{X}}\tilde{\text{b}} \end{array}$$

For a given number *K* of Gaussian kernels, their position is determined by k-means clustering in the voxel space and the covariance matrices of each cluster were estimated from the voxel positions within the corresponding clusters.

The number of smoothing kernels *K* in the three ROIs with 32, 82, and 126 voxels was set to 3, 4, and 8, respectively. The largest ROI is contained in both hemispheres. Hence, the sub-ROIs within each hemisphere were clustered independently into 4 clusters. Spatial smoothing with Gaussian kernels described above expresses the assumption that nearby voxels should have similar functionality. We refer to this approach as Spatial Grouping (SG) and to the resulting feature as SGF. An alternative approach would be to identify groups of voxels that are not only spatially close but also exhibit similarity in the activation time series (as quantified through cross-correlation) (Carpineto and Romano, 2012). We thus obtain *N* functional clusterings of the voxel space, one for each subject. These groupings at the subject level are then merged into a single population based functional clustering of voxels through Consensus Clustering (Carpineto and Romano, 2012). Given the resulting *K* voxel clusters, we calculated their means **μ**_*k*_ and covariance matrices **Σ**_*k*_, thus obtaining a set of *K* “functionally informed” smoothing Gaussian kernels $N\left({\text{r}}_{i};\mu_{k},\Sigma_{k}\right)$. The reduced graph matrix $\tilde{\text{X}}$ is then calculated as in Equatins (15) and (17). We refer to such functional voxel clustering as Functional grouping (FG) and to the resulting feature as FGF.

#### 3.1.3. Feature generation pipeline

**Figure 1:**
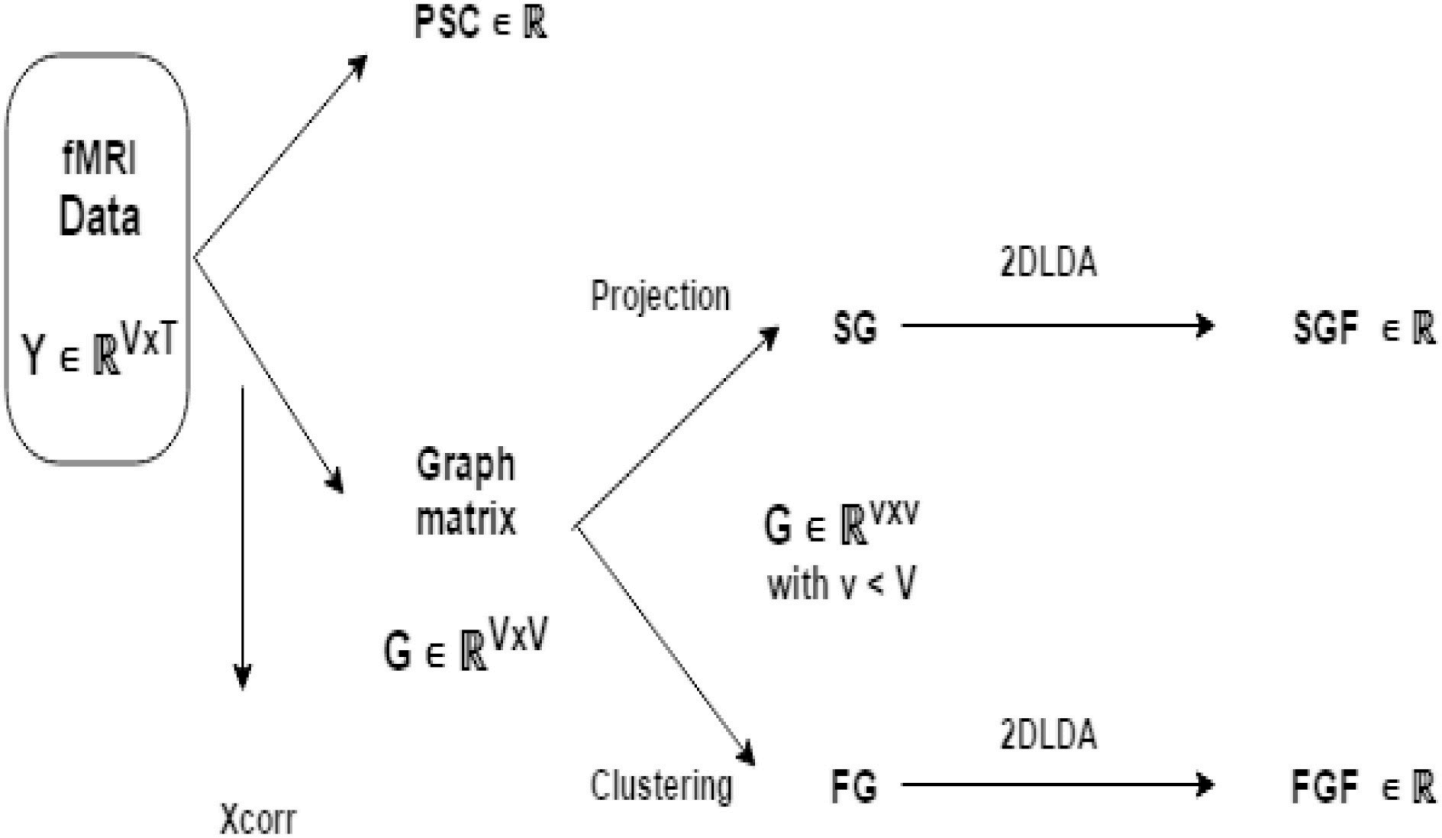
**Illustration of fMRI feature generation pipeline: from BOLD signal data Y to three fMRI features (PSC, FGF, and SGF)**. *F***G** and *S***G** are the reduced version of graph matrix **G** via functional grouping and spatial grouping (respectively). Note that FGF and SGF are both discriminative features extracted from *F***G** and *S***G** in a supervised manner using 2D-LDA (that is, Linear Discriminant Analysis operating on matrices).

Figure 1 illustrates the flow of fMRI feature generation. We obtain three fMRI features (PSC, FGF, SGF) independently from fMRI data **Y** ∈ ℝ^*V* × *T*^. Recall that *V* is number of voxels and *T* is the number of volumes. Feature *PSC* is computed directly from **Y**. To compute other two features, we first transform **Y** to a graph matrix **X** of size *V* × *V* and reduce **X** to $\tilde{\text{X}}$ of size *K* × *K* with (*K* < *V*) either through spatial projection or through functional clustering. Finally, we extract *SGF* from $\tilde{\text{X}}$ obtained by spatial projection and *FGP* from $\tilde{\text{X}}$ obtained by functional clustering.

### 3.2. Classification tools

#### 3.2.1. Generalized matrix learning vector quantization (GMLVQ)

The classification algorithms of Learning Vector Quantization (LVQ) (Arbib, 2003) are supervised learning paradigms which work iteratively to modify the quantization prototypes to find the boundaries of the class. LVQ classifiers are represented by a set of vectors, so-called prototypes, embodying classes in the input space, and a distance metric on the input data. During training, prototypes are adapted in an iterative manner to define class borders. For each training point, the algorithm determines two closest prototypes, one with the same class as the training point, and another with a different class. The position of the two closet prototypes are then updated, where same class prototype is moved closer to the data point, while different class prototype is pushed away from the data point. During testing, an unknown point is assigned to the class represented by the closest prototype with respect to the given distance.

The LVQ scheme, which is originally introduced by Kohonen et al. (2001), applies Hebbian online learning in order to adapt prototype with training data. Subsequent, researchers proposed a number of modifications to the basic learning scheme. Such variations utilize an explicit cost functionality, whereas others allow for incorporating adaptive distance measures (Schneider et al., 2009; Schneider, 2010).

Given training data $\left({\text{x}}_{i},y_{i}\right)\in {\mathbb{R}}^{m}\times \left\{1,\cdots ,K\right\},i=1,2,\cdots ,n$, where *m* denotes the dimensionality of data and *K* signifies the number of different classes. Typically, a LVQ network will include *L* prototypes ${\text{w}}_{q}\in {\mathbb{R}}^{m},q=1,2,3,\ldots ,L,$ which is characterized according to their location available in the input space and their class *c*(**w**_*q*_) ∈ {1, …, *K*}. At least one prototype in each class needs to be present. The overall number of prototypes is a model hyper-parameter that is to be optimized. The (squared) Euclidean distance *d*(**x**, **w**) = (**x** − **w**)^⊺^(**x** − **w**) within ℝ^*m*^ quantifies the distance between the input vectors and prototypes. The classification performed using the winner-takes all scheme: the data point ${\text{x}}_{i}\in {\mathbb{R}}^{m}$ belongs to the label *c*(**w**_*j*_) of the prototype **w**_*j*_ if and only if with *d*(**x**, **w**_*j*_) < *d*(**x**, **w**_*q*_), ∀*j* ≠ *q*. For every prototype **w**_*j*_ with class *c*(**w**_*j*_) a receptive field is defined within the input space. According to the LVQ model, points located in the respective field ^1^ will be assigned to the class *c*(**w**_*j*_).

The aim of learning is to adapt prototypes automatically in such a way that the gap between data points of class *c* ∈ {1, …, *K*} and the corresponding prototypes with label *c* (the one that the data are belonging to) will be reduced to a minimum distance. During the stage of training for each data point **x**_*i*_ with class label *c*(**x**_*i*_), the most proximal prototype with the same label is rewarded by pushing closer toward the training input; the most closest prototype with a different label will be disallowed by moving pattern **x**_*i*_ away.

The Generalized Matrix LVQ (GMLVQ) is a recent extension of the LVQ that employs a full matrix tensor for a better measure of distance between two feature vectors. The new distance measure not only is capable of scaling individual features but also accounts for pairwise correlations between the features. Assuming Λ ∈ ℝ^*m* × *m*^ is a positive definite matrix, Λ ≻ 0, the generalized form of the squared Euclidean distance is defined as

(15) $$\begin{array}{l} d_{\Lambda }\left({\text{x}}_{i},w\right)={\left({\text{x}}_{i}-\text{w}\right)}^{⊺}\Lambda \left({\text{x}}_{i}-\text{w}\right) \end{array}$$

The positive definiteness of Λ is guaranteed by imposing Λ = Ω^⊺^Ω, where Ω ∈ ℝ^*m* × *m*^ is a full-rank matrix. Furthermore, to prevent the degeneration of the algorithm, Λ is trace normalized after each learning step (i.e., $\underset{i}{\sum }\Lambda_{ii}=1$) so that the summation of eigenvalues is kept fixed in the learning process. The model is trained in an online-learning fashion and the steepest descent method is employed to minimize the cost function given as:

(16) $$\begin{array}{l} f_{GMLVQ}=\overset{n}{\underset{i=1}{\sum }}\phi \left(\mu_{\Lambda }\left({\text{x}}_{i}\right)\right) \end{array}$$

with

(17) $$\begin{array}{l} \mu_{\Lambda }\left({\text{x}}_{i}\right)=\frac{d_{\Lambda }\left({\text{x}}_{i},{\text{w}}^{+}\right)-d_{\Lambda }\left({\text{x}}_{i},{\text{w}}^{-}\right)}{d_{\Lambda }\left({\text{x}}_{i},{\text{w}}^{+}\right)+d_{\Lambda }\left({\text{x}}_{i},{\text{w}}^{-}\right)}, \end{array}$$

where ϕ is a monotonic function (the identity function ϕ(*l*) = *l* is a common choice). The main advantage of the GMLVQ framework is that (unlike LVQ, Schneider et al., 2009; Schneider, 2010), it allows us to naturally incorporate privileged information through metric learning.

#### 3.2.2. Privileged information (PI) guided GMLVQ

This paper employs the Information Theoretic Metric Learning (ITML) approach (Davis et al., 2007) in order to incorporate privileged information into the learning phase of the GMLVQ.

Given a training dataset, we have one space where the original training data live and another space where the privileged training data live. They are denoted by 𝒳 and 𝒳^*^, respectively, and their corresponding global metric tensors are denoted by **Λ** and Λ^*^. The distances between the privileged training points in 𝒳^*^ are first computed using Λ^*^ and then are sorted in ascending order. Based on the closeness information in 𝒳^*^, the original training points are tagged in a categorical manner (similar and dis-similar). After that, the ITML approach is adopted to impose similarity constraints in the original space. The main goal is to learn a new metric in the original space (denoted by Λ_new_) so that under the new metric, the distance between two original training points is small if their counterparts in the privileged space are similar (close), and vice versa. Implementation of the above concept is described in the following.

The training dataset is given as $\left\{\left({\text{x}}_{i},{\text{x}}_{i}^{*},y_{i}\right):{\text{x}}_{i}\in 𝒳,{\text{x}}_{i}^{*}\in {𝒳}^{*},i=1,2,\ldots ,N\right\}$. Recall that *y* represents class label. For each pair of two training examples, 1 ≤ *i* < *j* ≤ *N*, we compute three different squared Mahalanobis distances as follows

(18) $$\begin{array}{l} d_{\Lambda }\left({\text{x}}_{i},{\text{x}}_{j}\right)={\left({\text{x}}_{i}-{\text{x}}_{j}\right)}^{⊺}\Lambda \left({\text{x}}_{i}-{\text{x}}_{j}\right),{\text{x}}_{i},{\text{x}}_{j}\in 𝒳 \end{array}$$

(19) $$\begin{array}{l} d_{\Lambda^{*}}\left(x_{i}^{*},x_{j}^{*}\right)={\left(x_{i}^{*}-x_{j}^{*}\right)}^{⊺}\Lambda^{*}\left(x_{i}^{*}-x_{j}^{*}\right),x_{i}^{*},x_{j}^{*}\in {𝒳}^{*} \end{array}$$

(20) $$\begin{array}{l} d_{\Lambda_{\text{new}}}\left(x_{i},x_{j}\right)={\left(x_{i}-x_{j}\right)}^{⊺}\Lambda_{\text{new}}\left(x_{i}-x_{j}\right),x_{i},x_{j}\in 𝒳 \end{array}$$

Note that **Λ** and Λ^*^ are both given whereas Λ_new_ needs to be learned. The metric tensor Λ_new_ should be optimized in a supervised manner so that *d*_Λ_new__(**x**_*i*_, **x**_*j*_) will be shrunk if ${\text{x}}_{i}^{*}$ and ${\text{x}}_{j}^{*}$ are similar. Otherwise, *d*_Λ_new__(**x**_*i*_, **x**_*j*_) will be enlarged. To this end, we form two sets of pairs of the training data points in the original space 𝒳: *S*_+_ is a set of similar pairs and *S*_−_ a set of dissimilar pairs. These two sets are formed using the proximity information in the privileged space 𝒳^*^ as follows:

1. $\text{If }d_{\Lambda^{*}}$ $\left({\text{x}}_{i}^{*},{\text{x}}_{j}^{*}\right)$ $\leq l^{*}\text{ and }y_{i}=y_{j}$ (same class label), then (**x**_*i*_, **x**_*j*_) ∈ *S*_+_;
2. $\text{If }d_{\Lambda^{*}}$ $\left({\text{x}}_{i}^{*},{\text{x}}_{j}^{*}\right)$ $\geq u^{*}\text{ and }y_{i}\neq y_{j}$ (different class label), then (**x**_*i*_, **x**_*j*_) ∈ *S*_−_.

Here, l^*^ and u^*^ represent the upper and lower bound for the distances of similar and dissimilar pairs, respectively, in the privileged space. The value of l^*^ is chosen as the upper bound for the < a^*^ percentile of all $d_{\Lambda^{*}}(x_{i}^{*},x_{j}^{*})$ values, 1 ≤ *i* < *j* ≤ *N*. Similarly, the value of u^*^ is chosen as the lower bound for the > 1 − b^*^ percentile of all $d_{\Lambda^{*}}(x_{i}^{*},x_{j}^{*})$ values, 1 ≤ *i* < *j* ≤ *N*. At the same time, the choice of l^*^ and u^*^ is subject to the constraint u^*^ > l^*^. Also, a^*^ and b^*^ are pre-determined with 0 < a^*^ < b^*^ < 1.

In the GMLVQ framework, the privileged information is incorporated by fusing the metric Λ^*^ in the privileged space 𝒳^*^ with the metric **Λ** in the original space 𝒳 (for more details, see Fouad et al., 2013).

#### 3.2.3. Imbalanced class problem

Class imbalance occurs when there is a mismatch between sample sizes representing different classes. Class imbalance is one of the most common issues in classification. Unless explicitly treated, the classifier can be biased toward the majority class. In general, model fitting algorithms of various forms of classifiers assume balanced class distribution. A variety of methods have been proposed to tackle the class imbalance problem (e.g., García et al., 2007). For example, the imbalance problem can be addressed by either upsampling the minority class(es) (Pérez-Ortiz et al., 2015), or downsampling the majority class(es) (Elrahman and Abraham, 2013), so that the training set becomes balanced.

Since the data sets available for our study are relatively small, instead of upsampling small minority class, we decided to downsample the majority class, and repeat the downsampling *N*_*d*_ = 100 times. Training portion of the minority class remains fixed and each time the majority class is downsampled we construct a classifier based on balanced classes. We thus obtain a collection of *N*_*d*_ classifiers trained on different versions of downsampled majority class. These classifiers are then combined in an ensemble to form a single classifier using majority voting over the ensemble members.

#### 3.2.4. Employing different types of PI

We have two different kinds of features extracted from fMRI signals and used as privileged information, namely percent change (PSC) in overall ROI activation and graph based features described above.

The PSC feature quantifies the relative activation difference in the whole ROI when subjects were shown structured vs. random stimuli. This is calculated both from both pre- and post-training fMRI data. We consider 3 ROIs, hence there are 6 PSC privileged information features. Analogously, for the graph-based spatial-temporal features, there is a single feature for each ROI, measured both pre- and post-training, yielding a totality of 6 graph-based privileged information features.

An obvious combination of PSC and graph-based features would be to concatenate them into 12-dimensional vector. However, given the small sample size of participants, such an approach might lead to overfitting. Therefore we constructed an alternative way of combining privileged information features, as outlined below.

We independently construct two classifiers operating in the original space, but trained with the two different kinds of privileged information. Given a test input, if both classifiers predict the same class label, that label is used as the model output. If, on the other hand, they disagree, we output the class label that is predicted with “more confidence”—i.e., smaller distance between the test input and the closest class prototype.

However, note that for the classification purposes, the metric tensor in a single classifier can be arbitrarily scaled, since only the relative relations between distances of test point to the class prototypes are relevant. Hence, in order to compare distances of the test point to the closest prototype in the two classifiers, we need to normalize the learnt metrics. We do this by eigen-decomposing the two metric tensors Λ_1_ and Λ_2_ and normalizing their eigenvalues to sum to 1. In particular, the eigen-decomposition of Λ_*i*_, *i* = 1, 2, reads $\Lambda_{i}={\text{U}}_{i}\text{diag}\left(\lambda_{1}^{i},\lambda_{2}^{i},\ldots \lambda_{d}^{i}\right){\text{U}}_{i}^{⊺}$. The normalized metric tensor is obtained as

(21) $$\begin{array}{l} {\hat{\Lambda }}_{i}={\text{U}}_{i}\text{diag}\left({\hat{\lambda }}_{1}^{i},{\hat{\lambda }}_{2}^{i},\ldots ,{\hat{\lambda }}_{d}^{i}\right){\text{U}}_{i}^{⊺}, \end{array}$$

where the normalized eigenvalues are

(22) $$\begin{array}{l} {\hat{\lambda }}_{j}^{i}=\frac{\lambda_{j}^{i}}{\sum_{k=1}^{d}\lambda_{k}^{i}}. \end{array}$$

Given a test input, when combining two ensemble classifiers *C*_1_ and *C*_2_, if they agree on the predicted label, we output that label as the overall label estimate. If, however, *C*_1_ and *C*_2_ disagree on the label, we prefer the label produced with “more certainty”—in our context—small average distance to the closest prototype. In particular, if *C*_1_ is claiming class +1, we calculate the mean distance of the test input to the closest prototype of class +1 across those ensemble members that output class +1 (e.g., their closest prototype to the test input has label +1). Analogously, for *C*_2_ claiming class −1, we record the mean distance of the test input to the closest prototype of class −1 across ensemble members outputting class −1. The overall class label of the combined classifier for the test input is the label with the minimal average distance to the closest prototype.

### 3.3. Experimental design

The value of using brain imaging data as privileged information in our setting can be evaluated through two extreme cases:

- No privileged information is available—the models (classifiers) are constructed purely based on the cognitive data. We will refer to this case as *M*-CD;
- Privileged brain imaging data is always available and is used directly as input data in the classifier construction and testing, without the need to resort to learning with privileged information. We will refer to this case as *M*-PD. The classifiers obtained in this regime with the PSC, FGF, and SGF representations of brain imaging data are referred to as *M*-PSC, *M*-FGF, and *M*-SGF, respectively.

When the classifiers are constructed in the framework of learning with privileged information, with cognitive data serving as classifier inputs and brain imaging data used as privileged information, depending on what representation of brain imaging data is used, we denote the resulting classifiers by *M*^+^-CD-PSC, *M*^+^-CD-FGF, and *M*^+^-CD-SGF.

**Figure 2:**
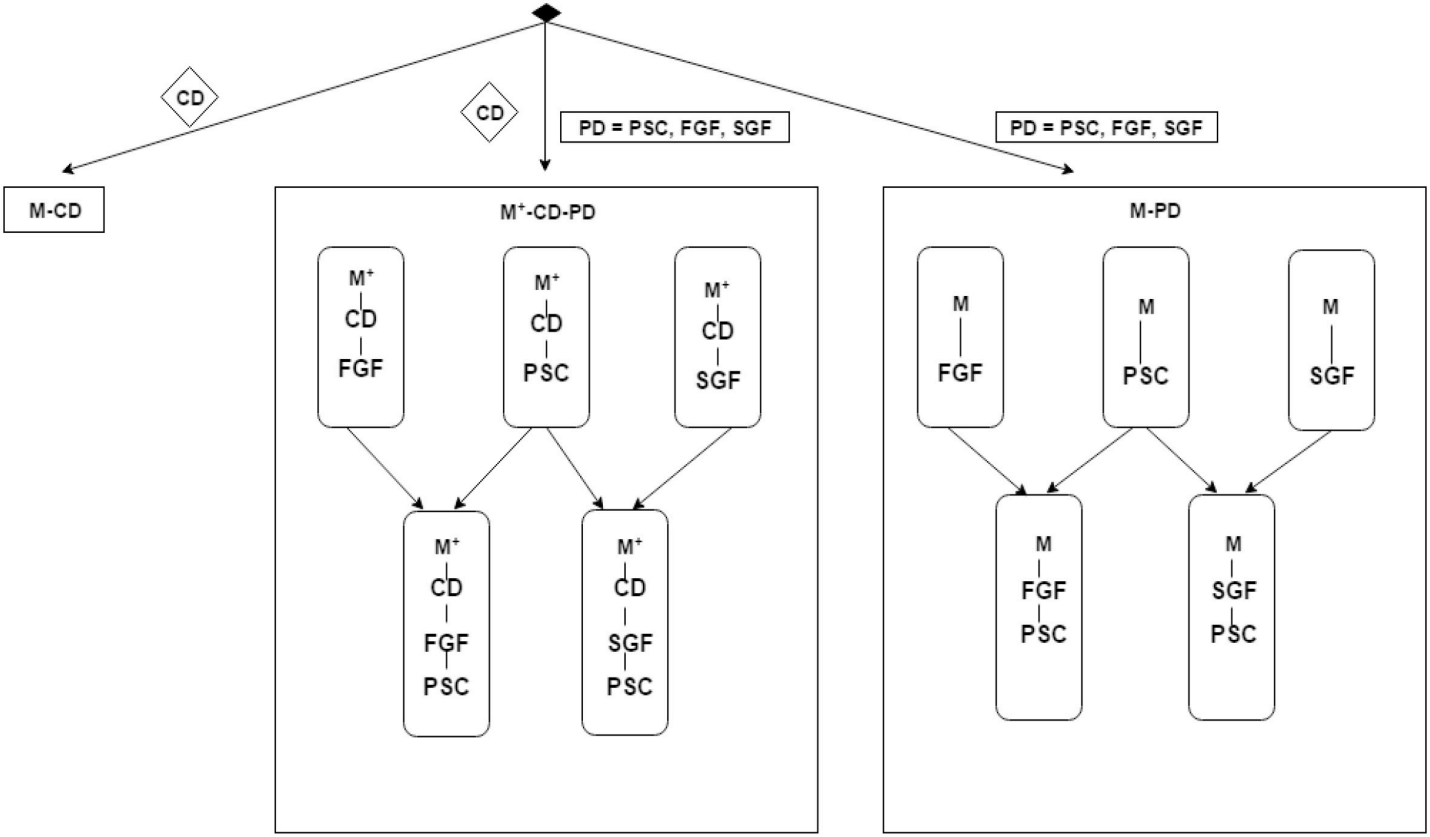
**Schematic illustration of the experimental design described in Section 3.3**. The items in diamond shape denote data: CD for cognitive data, PD for privileged information data, PSC for Percent Signal Change, FGF for functionally grouped graph feature, and SGF for spatially grouped graph feature. *M*-XXX denotes a GMLVQ classifier that does not use privileged information while XXX denotes the inputs to this classifier. For example, *M*-PSC means a GMLVQ classifier with PSC features as its inputs. *M*^+^-XXX-YYY denotes a GMLVQ classifier using feature XXX as its inputs and feature YYY as privileged information. For example, *M*^+^-CD-PSC means a GMLVQ classifier using cognitive features as its inputs and PSC features as privileged information. *M*^+^-XXX-YYY-ZZZ denotes a hybrid classifier that combines the classification output of classifier *M*^+^-XXX-YYY and classifier *M*^+^-XXX-ZZZ using a certain rule (e.g., majority voting rule).

As explained above, PSC representation of spatial-temporal structure of cortical activations within an ROI is the simplest one, integrating out both the spatial and temporal structures. In contrast, a more subtle representation is obtained in the graph based features FGF and SGF, integrating over time, but preserving aspects spatial structure. The PSC and graph based features may contain complementary information for the classification task and hence we further combine the classifiers obtained using brain imaging data into composite ones, in particular *M*^+^-CD-PSC and *M*^+^-CD-FGF are combined into a single classifier *M*^+^-CD-PSC+FGF and the combination of *M*^+^-CD-PSC and *M*^+^-CD-SGF is referred to as *M*^+^-CD-PSC+SGF. Analogously, *M*-PD-PSC and *M*-FGF are combined to form *M*-PSC+FGF and combination of *M*-PSC with *M*-SGF results in *M*-PSC+SGF. The overall model structure setup is illustrated in Figure 2.

## 4. Experiments

This section assesses the classification performance of the proposed methodology that incorporates fMRI as privileged information (PD) in the training phase, against baseline algorithms trained without PD, or trained solely with PD. Since we expect that the brain imaging fMRI data carry lot of information regarding possible MCI, the classier trained directly on fMRI (M-PD) will provide a lower bound on the classification error that a classifier trained solely on cognitive data (M-CD) (carrying less information on possible MCI) cannot achieve. We expect that the power of learning with privileged information will boost the classification performance, so that the classifier trained with CD as inputs, but able to incorporate fMRI indirectly in the training process (*M*^+^-CD-PD), will have classification performance between the two extremes M-PD and M-CD, even though in the test phase, both M-CD and *M*^+^-CD-PD classify solely based on CD. The methodology is formulated in the framework of prototype-based classification (GMLVQ) with metric learning (Schneider et al., 2009; Schneider, 2010; Fouad et al., 2013). In this experiment, the original and privileged features correspond to cognitive profiles and brain imaging data, respectively. The overall experimental design is explained in Section 3.3.

### 4.1. Experimental setup

In the *M*-PD case, we have in total a set of 34 subjects having both cognitive and brain imaging data, consisting of 9 patients and 25 controls. We create 50 training-test set splits by randomly sampling 6 and 17 patients and controls, respectively, to form the training set (the rest is in the test set). In the *M*-CD case we have 60 subjects having cognitive data, consisting 13 patients and 47 controls. Again, we created 50 training-test set splits by randomly sampling 9 and 33 patients and controls, respectively, to form the training set. We made sure that in each resampled training and test set there is an equal balance between subjects with and without PD.

As explained in Section 3.2.3, to deal with class imbalance in the M-PD case, we construct ensemble classifiers by using the same set of 6 patients and repeatedly sampled 6 controls from the 17 training ones. Analogous setting was used in the M-CD case, this time with 9 patients and 33 controls.

In all experiments, the (hyper-)parameters of the ensemble classifiers were tuned via cross-validation on the training set of the first sub-split only. The found values were then fixed across the remaining 99 classifiers. In the GMLVQ classifier, data classes are represented by one prototype per class. The class prototypes are initialized as means of random subsets of training samples selected from the corresponding class. In the IT metric learning settings given in Fouad et al. (2013), lower (a, a^*^) and upper (b, b^*^) percentile bounds for the privileged and original spaces were tuned over the values of 5, 10, 15 and of 85, 90, 95, respectively.

Throughout the experiments we had one data set in the original space of CD. However, experiments were repeated for three different fMRI PD: PSC, SGF, and FGF. PD of each subject is represented by 6 features, 3 pre-training, and 3 post-training, corresponding to 3 ROIs. Due to the imbalanced nature of our classes we utilized the following below evaluation measures:

*1. Confusion Matrix*: it is a popular performance indicator for machine learning algorithms. It is organized along the actual classes (rows) and the predicted ones columns (Elrahman and Abraham, 2013). In this study positive and negative examples represent patients and controls, respectively. In the confusion matrix, True Positive (TP) denotes the number of positive examples correctly classified, True Negatives (TN) is the number of negative examples correctly classified, False Positives (FP) is the number of negative examples incorrectly classified, False Negatives (FN) is the number of positive examples incorrectly classified as negative. The true positive rate ($TPR=\frac{TP}{TP+FN}$) measures the percentage of patients who are correctly classified, whereas the true negative rate ($TNR=\frac{TN}{TN+FP}$) measures the proportion of the correctly identified controls. False positive rate ($FPR=\frac{FP}{FP+TN}$), refers to the probability of falsely classifying the patients, whereas the false negative rate ($FNR=\frac{FN}{FN+TP}$) refers to the probability of falsely classifying the controls. *2. Macroaveraged Mean Absolute Error (MMAE)*: it is a macroaveraged version of Mean Absolute Error and it is a weighted sum of the classification errors across classes (Fouad, 2013). It measures the per-class accuracy of class predictions ŷ with respect to true class *y* on a test set:

(23) $$\begin{array}{l} MMAE=\frac{1}{N}\sum_{n=1}^{N}\frac{\sum_{y_{i}=N}\left|y_{i}-{\hat{y}}_{i}\right|}{T_{n}} \end{array}$$

Where *N* is the number of classes and *T*_*n*_ is the number of test points whose true class is *n*.

### 4.2. Classification results

We are primarily interested in classification performance of *M*^+^-CD-PD classifiers, that is, classifiers using cognitive data as their inputs and incorporating brain imaging data as privileged information. this classification performance will be put in the context of performances when no brain imaging information is available (*M*-CD) and when the full brain imaging is available as input (*M*-PD). This will allow us to quantitatively investigate how much performance improvement over *M*-CD could be obtained by incorporating privileged information through metric learning. Following our experimental setup, we obtained 50 MMAE estimates for each classifier summarized by the mean, standard deviation, median and the (25%, 75%) percentiles. The results are summarized in Tables 1, 2.

**Table 1:** **Classification performance measured by Macroaveraged Mean Absolute Error (MMAE) for the baseline classifier, ***M***-CD, and five different ***M***-PD classifiers (see Column 1)**.

**Models**	**Mean**	**Std-Dev**	**Median**	**(25%, 75%) Percentile**	***p*-value**
*M*-CD	0.3992	0.0949	0.3942	(0.3173, 0.4423)	–
*M*-PSC	0.2357	0.1655	0.2381	(0.1429, 0.3333)	0.00
*M*-SGF	0.2666	0.1151	0.2995	(0.2143, 0.4048)	0.00
*M*-FGF	0.2376	0.1231	0.2381	(0.1429, 0.3333)	0.00
*M*-PSC+SGF	0.2438	0.1067	0.2381	(0.2143, 0.3095)	0.00
*M*-PSC+FGF	0.2200	0.1245	0.2143	(0.1429, 0.3095)	0.00

*For each classifier, we report both mean MMAE, its standard deviation, median MMAE and its (25%, 75%) percentile in Column 2 – 5, respectively. They were computed using the MMAE estimates obtained from 50 randomly created training-test splits. Each p-value displayed in Column 6 was obtained from one-sided sign-rank test against the null hypothesis that the corresponding M-PD classifier is inferior to the baseline classifier*.

**Table 2:** **The same as in Table 1 but for evaluation of the classification performance of five different ***M***^**+**^-CD-PD classifiers, that is, the classifiers using CD as their inputs and PD as privileged information**.

**Models**	**Mean**	**Std-Dev**	**Median**	**(25%, 75%) Percentile**	***p*-value**
*M*^+^-CD-PSC	0.3448	0.0988	0.3173	(0.2788, 0.4038)	0.00
*M*^+^-CD-SGF	0.3128	0.0804	0.3153	(0.2308, 0.3942)	0.56
*M*^+^-CD-FGF	0.3925	0.1211	0.3810	(0.2885, 0.4135)	0.12
*M*^+^-CD-PSC+SGF	0.3426	0.1116	0.3558	(0.2788, 0.4038)	0.00
*M*^+^-CD-PSC+FGF	0.3553	0.1157	0.3413	(0.2788, 0.4808)	0.04

Table 1 shows that for all five types of PD, *M*-PD outperforms *M*-CD. Recall that we have extracted three different features from the brain imaging data, namely PSC, SGF, and FGF, and all of them can be used as PD. For PSC, which is related to brain activation level, the corresponding median MMAE is reduced by relatively 39.6% when compared to that of *M*-CD. The other two types of PD, SGF, and FGF, are related to brain connectivity pattern. When compared to the baseline classifier, the relative reduction of their median MMAE is about 24 and 40%, respectively. The above results indicate that PSC is at least as useful as the graph feature (FGF), or even more useful (SGF). Im principle, the activation level and connectivity pattern are two independent fMRI features. Therefore, PSC could be used as PD along with SGF or FGF. Row 6–7 in Table 1 show that the resulting classifier can either attain the classification performance of *M*-PSC in the case of SGF, or improve on it in the case of FGF. In summary, brain imaging data contain more information that are relevant to the task than cognitive data.

Table 2 shows that for all five types of PD, *M*^+^-CD-PD outperforms *M*-CD. In particular, PSC and SGF are the best two among the five PD types that are used as the privileged information along with CD as GMLVQ's inputs. Compared to *M*-CD, both *M*^+^-CD-PSC + *M*^+^-CD-SGF show a reduction of their median MMAE by relatively 20%. This relative improvement is shrunk to 13.4, 9.7, and 3.4% for *M*^+^-PSC+FGF, for *M*^+^-PSC+SGF, and for *M*^+^-FGF (respectively).

Table 3 presents the results of average TPR and TNR of the models. The best two TPR results (0.83 and 0.80) were achieved by *M*-SGF and *M*-PSC-SGF (respectively), whereas the best two TNR result (0.88 and 0.87) were attained by *M*-PSC and *M*^+^-CD-FGF (respectively). Overall, *M*-FGF emerges as the classifier with most balance performance.

**Table 3:** **Overall true positive rates (TPR) and true negative rates (TNR) on hold-out sets**.

**Model**	**TPR**	**TNR**
*M*-CD	0.60	0.60
*M*-PSC	0.64	0.88
*M*^+^-CD-PSC	0.69	0.63
*M*-SGF	0.83	0.51
*M*^+^-CD-SGF	0.53	0.67
*M*-PSC+SGF	0.80	0.68
*M*^+^-CD-PSC+SGF	0.56	0.70
*M*-FGF	0.74	0.76
*M*^+^-CD-FGF	0.38	0.87
*M*-PSC+FGF	0.56	0.70
*M*^+^-CD-PSC+FGF	0.61	0.70

### 4.3. Further analysis

GMLVQ is a fully adaptive algorithm to learn global metric tensor which accounts for different importance weighting of individual features and pairwise interplay between the features, with respect to the given classification task. Hence, it allows us to study the task-dependent relevance of the input features by using the diagonal elements of the GMLVQ metric tensor matrix. Moreover, the global metric can be further optimized adaptively by incorporating privileged information into the GMLVQ model via the distance relations revealed in the privileged space (Fouad, 2013). In the following we analyse the learned classification models in terms of the learned metric tensor and discuss possible implications regarding the cognitive and brain imaging fMRI features used in this study.

#### 4.3.1. Cognitive features only

We first present a procedure to study the relevance of four cognitive features (working memory, cognitive inhibition, divided attention, and selective attention) using the GMLVQ metric (tensor) matrices obtained from the experiments whose classification results are discussed in Section 4.2. Each of these experiments resulted in 50 × 100 GMLVQ classifiers with the associated metric (tensor) matrices Λ obtained by training GMLVQ classifiers on 50 × 100 (small) data sets independently. Recall that these data sets were generated by first randomly splitting the whole training set into 50 smaller sets of equal size and then randomly downsampling the majority class to the size of the minority class in each split 100 times. However, many of the 50 × 100 classifiers performed poorly and they should not be included in the analysis of the relevant cognitive features. We therefore discard the data split producing the ensemble classifier whose *N*_*b*_-th best ensemble member (classifier) produced error larger than a threshold value denoted by *E*_*max*_, and pool all ensemble members from each of the remaining splits for further analysis. This procedure is applied to three experiments as follows: *M*-CD, *M*^+^-CD-PSC and *M*^+^-CD-FGF. We found out that *N*_*b*_ = 15 and *E*_*max*_ = 25% worked universally across these data sets.

**Figure 3:**
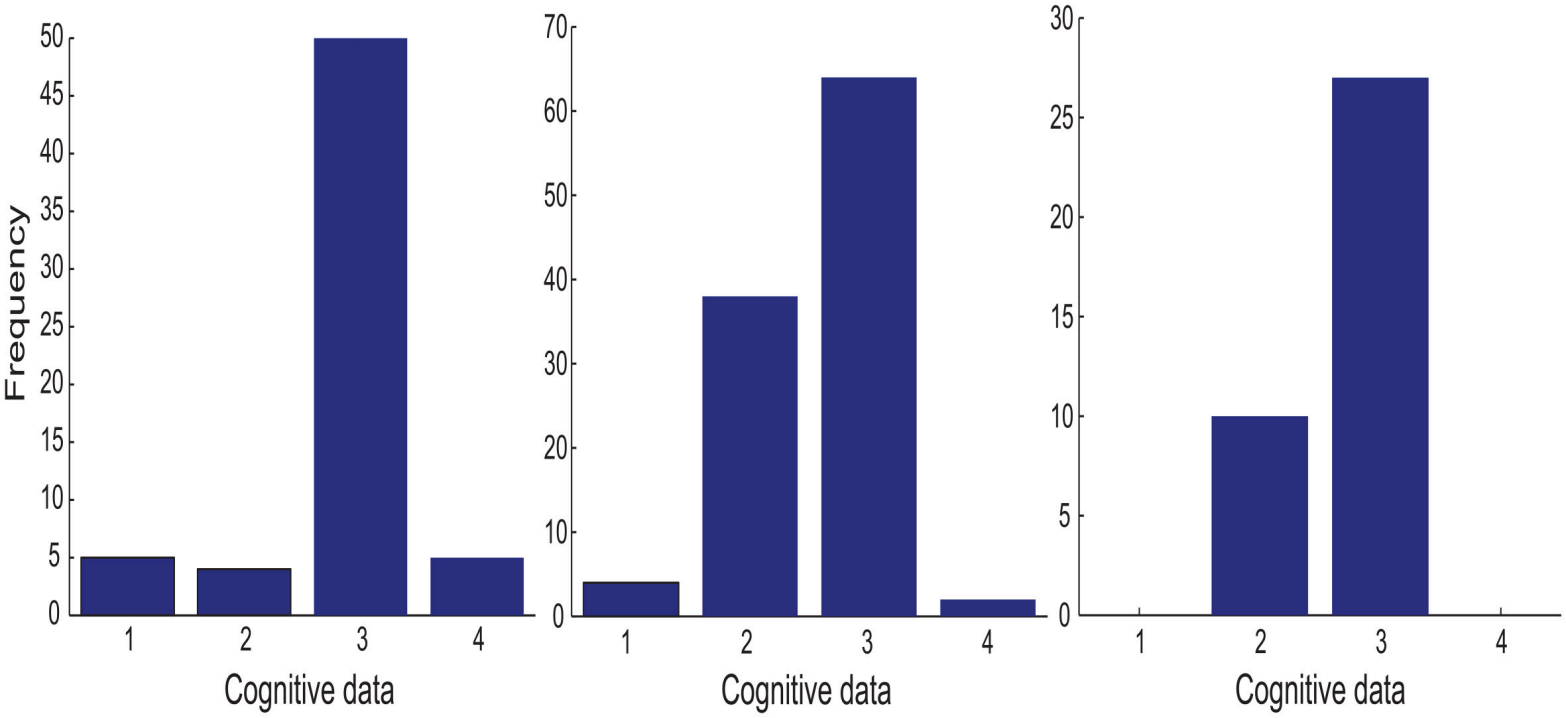
**The importance histogram of the four cognitive features as follows: working memory (***n***_**dots**_), cognitive inhibition (***t***_**delay**_), divided attention ($t_{\text{disp}}^{d}$), and selective attention ($t_{\text{disp}}^{s}$) (numbered as 1, 2, 3, and 4 in the order)**. These features are used as the input to the following GMLVQ classifiers: *M*-CD, *M*^+^-CD-PSC, and *M*^+^-CD-FGF (from left to right). Note that each cognitive feature is associated with a diagonal element of the GMLVQ metric tensor matrix Λ and the importance histogram counts the number of each diagonal element in the >90% percentile of all diagonal elements from an ensemble of Λs.

Each of the four cognitive features is associated with one of the four diagonal element in the metric (tensor) matrix. For each cognitive feature, its importance is measured by the frequency of its associated diagonal elements in >90% percentile of the set of all diagonal elements from the metric (tensor) matrices selected by the above procedure. The left panel in Figure 3 shows that the divided attention (i.e., $t_{\text{disp}}^{d}$) is the most discriminative feature for the classification task (MCI patients vs. healthy controls).

Next, we studied the off-diagonal elements of those metric (tensor) matrices. Each off-diagonal element controls the interplay between two associated cognitive features. To illustrate how this interplay works, we provide a toy example as follows: Denote a two-dimensional feature vector by (*x*, *y*) and a 2 × 2 metric tensor by $\left(\begin{array}{cc} \alpha & \gamma \\ \gamma & \beta \end{array}\right)$. The distance between two feature vectors indexed by *i* and *j* is given by

(24) $$\begin{array}{l} d_{ij}=\underset{d_{ij}^{M}}{\underset{︸}{\alpha^{2}\cdot {\left(x_{i}-x_{j}\right)}^{2}+\beta^{2}\cdot {\left(y_{i}-y_{j}\right)}^{2}}}+\underset{d_{ij}^{2}}{\underset{︸}{2\gamma \cdot (x_{i}-x_{j})(y_{i}-y_{j})}}. \end{array}$$

The first two terms of *d*_*ij*_ is actually so-called Mahalanobis distance between the *i*-th and *j*-th feature vectors (denoted by $d_{ij}^{M}$). In the case of γ = 0, the diagonal term α and β are optimized by maximizing between-class Mahalanobis distances while minimizing within-class ones. When the metric matrix has non-zero off-diagonal elements, the distance measure has additional contribution $d_{ij}^{2}$ which can either enhance or collapse the total distance measure depending on *(i)* the sign of γ and *(ii)* the sign of *between-class* correlation (i.e., correlation between class-conditional means of *x* and *y*). For example, in the case of negative *between-class* correlation, negative γ can further enhance the class separation and vice versa.

**Figure 4:**
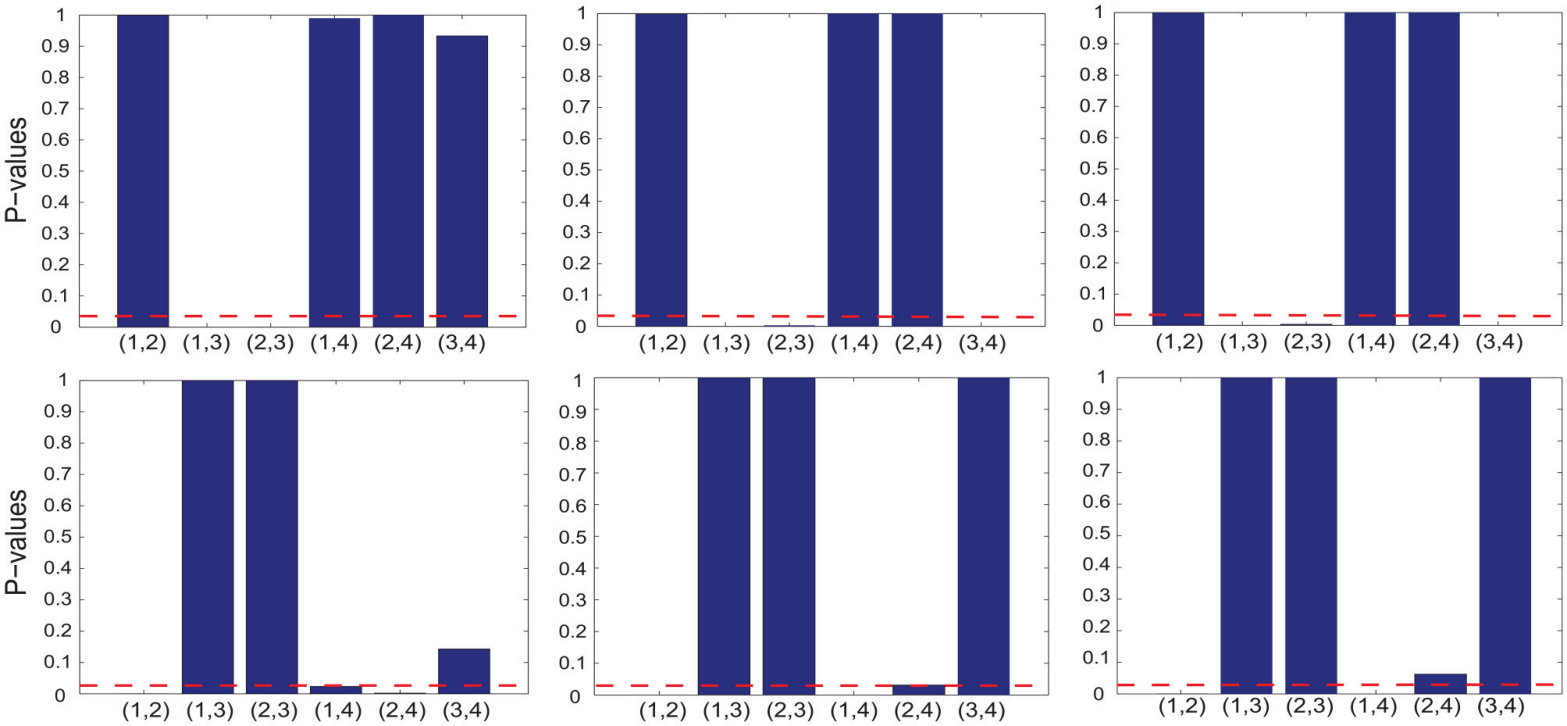
**The ***p*** values of the one-sided sign-rank tests for studying the interplay between two of the following cognitive features: working memory (***n***_**dots**_), cognitive inhibition (***t***_**delay**_), divided attention ($t_{\text{disp}}^{d}$), and selective attention ($t_{\text{disp}}^{s}$) (numbered as 1, 2, 3, and 4 in the order)**. From each panel in the upper and lower row, one can read that if the *p* value is smaller than the threshold *p* = 0.05 (indicated by red dashed line), the interplay of two corresponding cognitive features is statistically significant and it takes a negative and positive value (respectively); These features are the inputs to three GMLVQ classifiers as follows: *M*-CD, *M*^+^-CD-PSC, and *M*^+^-CD-FGF (from left to right). Note that the tests used the off-diagonal elements of the GMLVQ metric tensor matrices.

To test whether the interplay between two cognitive features, indexed by *i* and *j*, is positive or negative, we performed two one-sided sign-rank tests for the hypotheses Λ_*ij*_ > 0 and Λ_*ij*_ < 0 (respectively) using the corresponding off-diagonal element from the selected GMLVQ metric (tensor) matrices. The upper-left panel of Figure 4 shows that there exists statistically significant, negative interplay between divided attention and two following cognitive features: (1) working memory (*n*_dots_) and (2) cognitive inhibition (*t*_delay_). From the lower-left panel, we found statistically significant, positive interplay between three cognitive features as follows: (1) working memory, (2) cognitive inhibition, and (3) selective attention ($t_{\text{disp}}^{s}$). Finally, note that there is no significant interplay between divided attention and selective attention.

**Figure 5:**
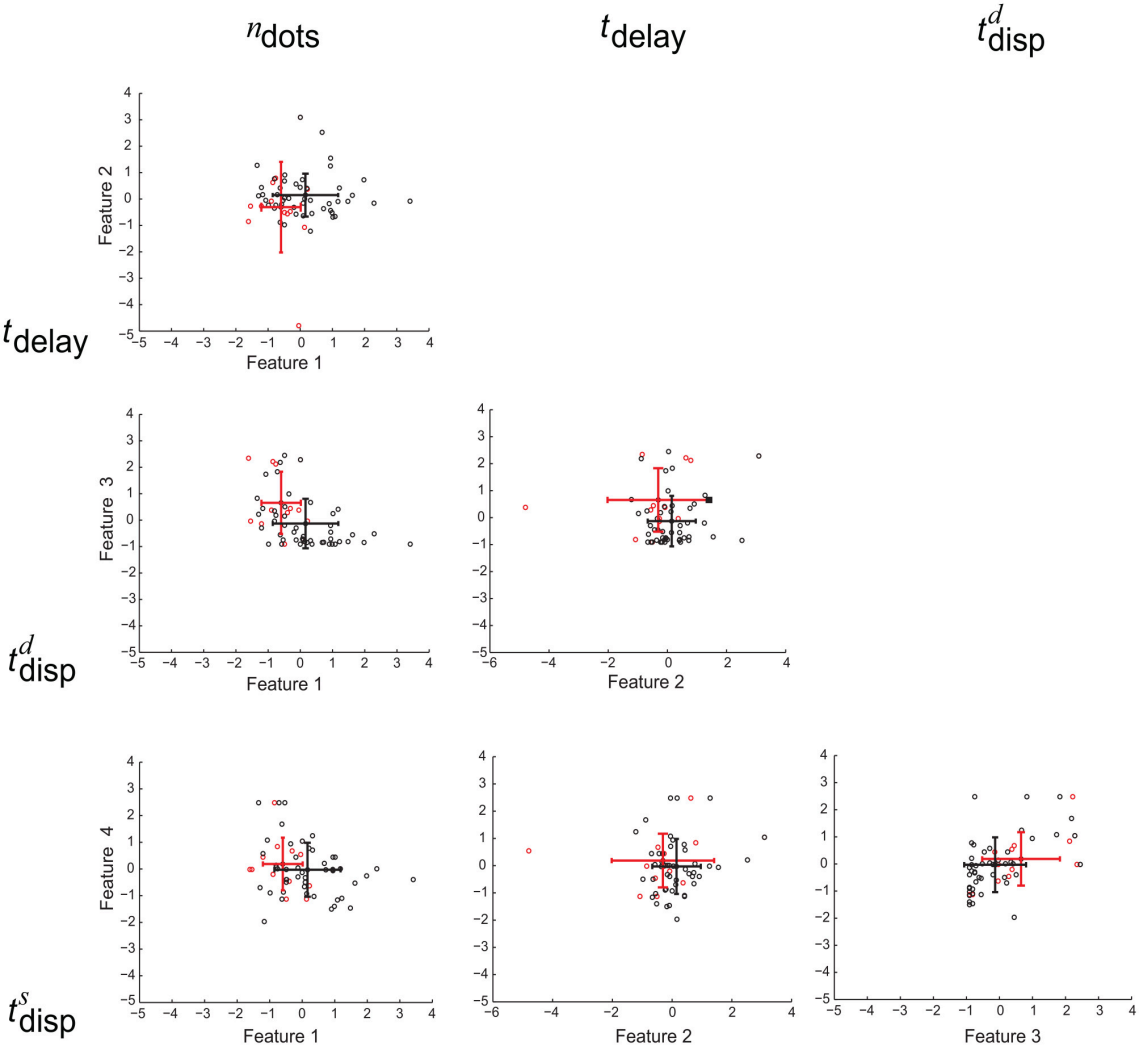
**Scatter plot for six possible feature pairs from the four cognitive features as follows: working memory (***n***_**dots**_), cognitive inhibition (***t***_**delay**_), divided attention ($t_{\text{disp}}^{d}$), and selective attention ($t_{\text{disp}}^{s}$) (numbered as 1, 2, 3, and 4 in the order)**. For individual MCI patients and healthy controls, their feature pairs (i.e., Feature 1 vs. Feature 2) are displayed as red and black dots (respectively). The corresponding class-conditional means and standard deviations are also displayed by colored error bars. For each panel, the corresponding two features are indicated at the top of each column and on the utmost left of each row (respectively).

To examine the relation between the interplay and *between-class* correlation revealed by Equation (24), we need to determine whether or not there exists statistically significant *between-class* correlation between two of the four cognitive features. To this end, we first used one-sided sign-rank test to determine, for each of the four features, whether its values for MCI patients are significantly larger or significantly smaller than those for healthy controls. For each pair of the cognitive features, if the outcomes of their tests are both statistically significant and are consistent with (or in opposite to) each other, then their *between-class correlation* is considered as positive (or negative). Otherwise, the *between-class* correlation is insignificant. From this analysis we observe (1) the class-conditional mean of working memory is positively correlated with that of cognitive inhibition; and (2) the class-conditional mean of divided attention is negatively correlated with that of working memory as well as that of cognitive inhibition. These observations agree with the observation of the interplay between the corresponding cognitive features, which can enhance the class separation. For the remaining pairs of the cognitive features, their *between-class correlation* is not significant. In Figure 5, we graphically illustrate the presence or absence of these correlations.

In summary, though the divided attention is the most relevant feature among the four cognitive features, all four features are indispensable for maximizing the classification performance. This is because these exists *between-class correlation* between the features.

#### 4.3.2. fMRI features

We carried out the same relevance analysis for *M*-PSC, *M*-SGF, and *M*-FGF as for *M*-CD in Section 4.3.1. Recall that in these three experiments, the inputs to GMLVQ classifiers are comprised of six fMRI features as follows: (i) PSC-Cerebellar-Pre, PSC-Cerebellar-Post, PSC-Frontal-Pre, PSC-Frontal-Post, PSC-Subcortical-Pre, PSC-Subcortical-Post; (ii) SGF-Cerebellar-Pre, SGF-Cerebellar-Post, SGF-Frontal-Pre, SGF-Frontal-Post, SGF-Subcortical-Pre, SGF-Subcortical-Post; and (iii) FGF-Cerebellar-Pre, FGF-Cerebellar-Post, FGF-Frontal-Pre, FGF-Frontal-Post, FGF-Subcortical-Pre, FGF-Subcortical-Post (respectively). The fMRI feature “PSC-Cerebellar-Pre” denotes PSC feature that is derived from fMRI data measured in the cerebellar ROI and during the pre-training session. and the remaining fMRI features are abbreviated in the same way. Recall that PSC is referred to as Percent Signal Change, SGF as Spatially grouped Graph Feature and FGF as Functionally grouped Graph Feature.

**Figure 6:**
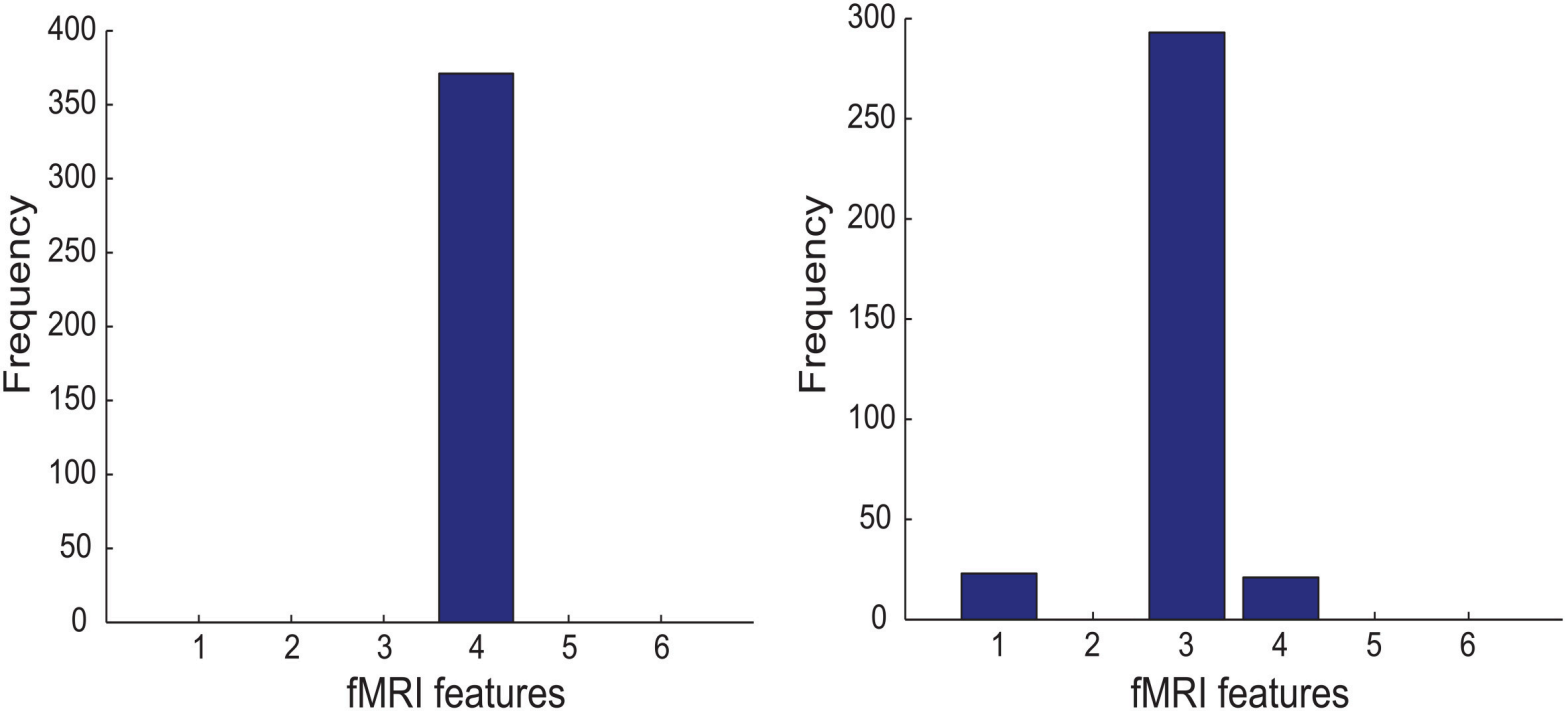
**Left panel:** The importance histogram of the six fMRI features as follows: PSC-Cerebellar-Pre, PSC-Cerebellar-Post, PSC-Frontal-Pre, PSC-Frontal-Post, PSC-Subcortical-Pre, and PSC-Subcortical-Post. (numbered as 1, …, and 6 in the order). PSC is referred to as Percent Signal Change, Pre as Pre-training session, Post as Post-training session, Cerebellar (Frontal and Subcortical) as the cerebellar(frontal and subcortical, respectively) ROI. For example, PSC-Cerebellar-Pre means that the fMRI data were acquired before training and PSC feature was extracted from the cerebellar ROI). **Right panel:** The same as in the left panel but for the following fMRI features: FGF-Cerebellar-Pre, FGF-Cerebellar-Post, FGF-Frontal-Pre, FGF-Frontal-Post, FGF-Subcortical-Pre, and FGF-Subcortical-Post.

Figure 6 shows that PSC-Frontal-Post and FGF-Frontal-Pre are the most discriminative fMRI feature in Experiment *M*-PSC and *M*-FGF (respectively). We first note that the most relevant feature in both cases is derived from the frontal ROI (that is, the largest ROI among the three ROIs used in this study). It is more interesting to address two following questions: (1) why is the post-training session is more relevant than the pre-training one, when PSC is used for the task; and (2) why is the opposite true when the graph feature is used for the task.

**Figure 7:**
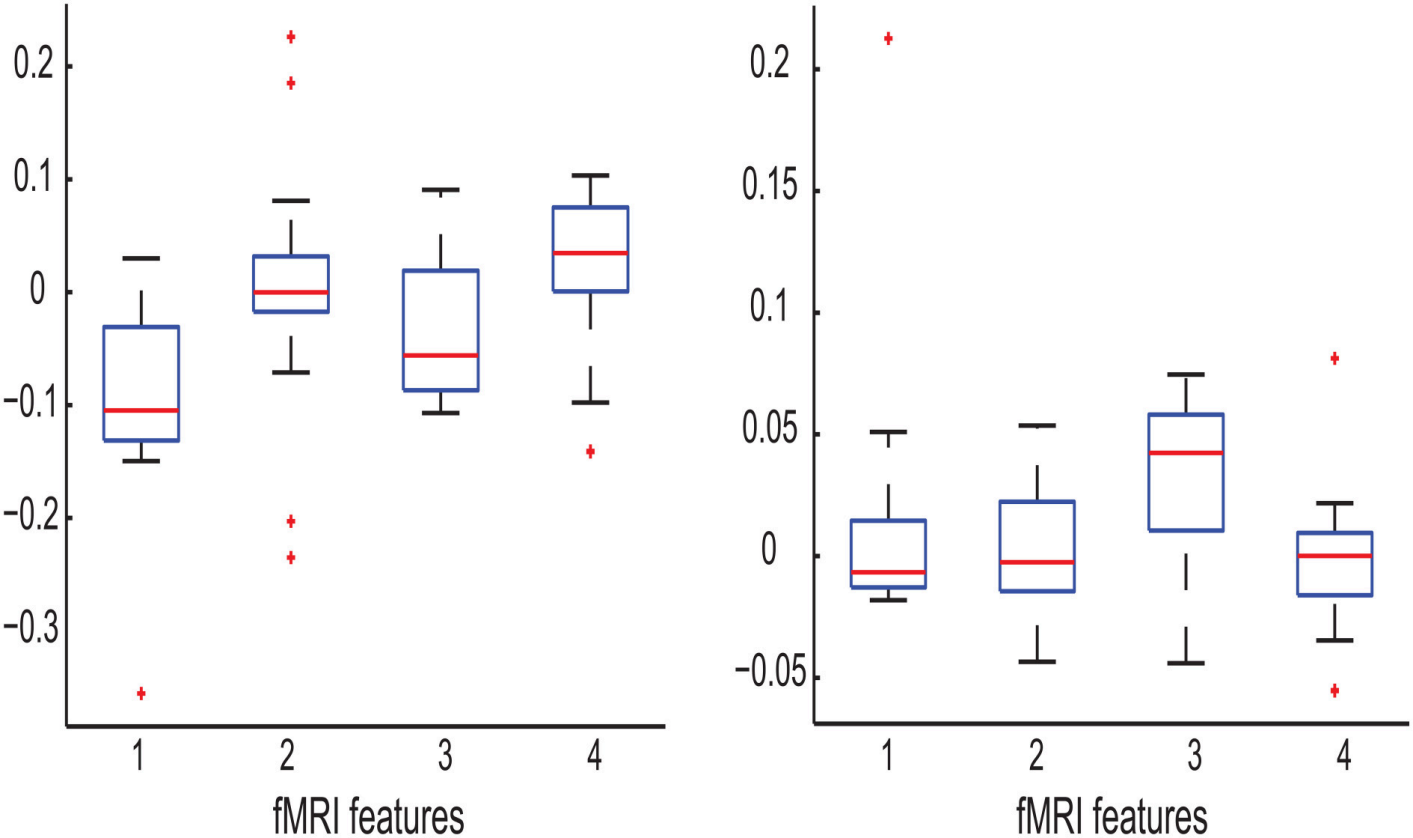
**Left:** Boxplot of the following fMRI features: FGF-Frontal-Pre for MCI patients, FGF-Frontal-Pre for healthy controls, FGF-Frontal-Post for MCI patients, and FGF-Frontal-Post for healthy controls (numbered as 1, 2, 3, and 4 in the order). Note that the *y*-axis represents the values of the corresponding fMRI features; **Right:** Boxplot of the following fMRI features: PSC-Frontal-Pre for MCI patients, PSC-Frontal-Pre for healthy controls, PSC-Frontal-Post for MCI patients, and PSC-Frontal-Post for healthy controls (numbered as 1, 2, 3, and 4 in the order).

The left panel in Figure 7 shows that before training, the PSC level for MCI patients and healthy controls are on average comparable. However, training caused a remarkable increase of the PSC level for MCI patients but not for healthy controls. As a result, these two participant groups differ in their PSC level after the training. This is why PSC-Frontal-Post is identified as the most relevant feature for Experiment *M*-PSC. The right panel in Figure 7 shows that the graph feature FGF differs between MCI patients and healthy controls before training. This could be related to the suggestions that MCI may have caused changes in brain connectivity. We further observe that for both participant groups, training increased their FGF values but to different extents. After training, the difference between MCI patients and healthy controls became much less significant. This is why FGF-Frontal-Pre is identified as the most relevant feature for Experiment *M*-FGF. This observation allows us to speculate that training could “mitigate” the changes in brain connectivity caused by MCI.

**Figure 8:**
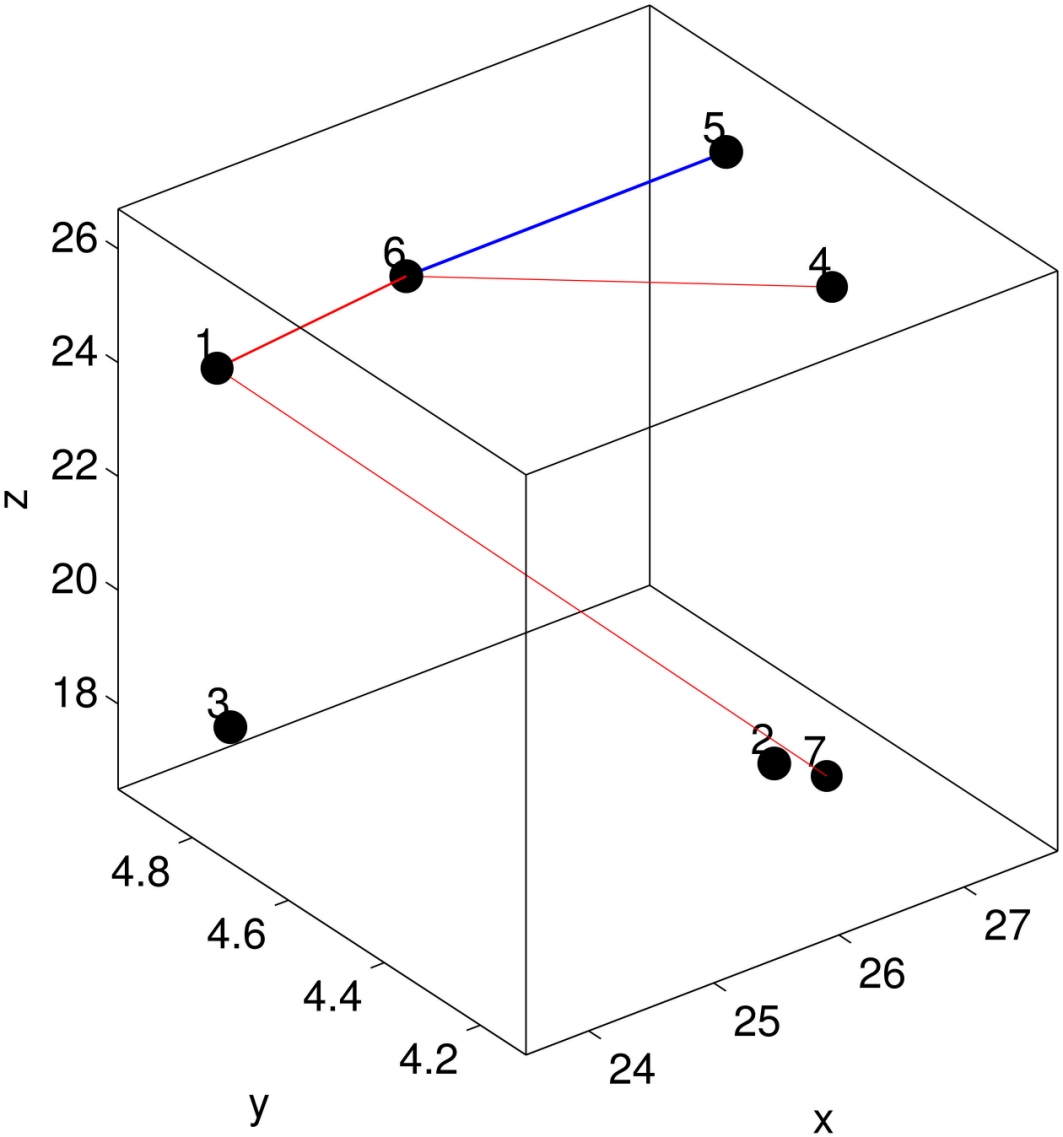
**The node configuration for the frontal ROI which includes Superior Frontal Gyrus on the right hemisphere and Medial Frontal Gyrus on the left hemisphere**. The straight lines indicate the edges whose importance for discriminating MCI patients from healthy controls has significantly changed. For the three-node subnetwork (indicated by red lines), its importance has increased after training. In contrast, the single-node subnetwork (indicated by blue line), training has reduced its importance.

The above analysis suggests that brain connectivity may have changed after training and this is significant particularly for MCI patients. In the following, we address the question whether a sub-network rather than the entire (local) network within the frontal ROI has changed. Recall that all 128 voxels in the frontal ROI are grouped into 7 spatially contiguous clusters. This results in a local brain network consisting of 7 nodes and 21 edges (see Figure 8). Each off-diagonal element of the graph matrix *G* quantifies the connectivity between two nodes and measures the strength of the corresponding edge. Recall that the graph features FGF were extracted by applying 2D-LDA. To this end, 2D-LDA provides two feature-generating vectors **a** and **b** from which we can derive a task-dependent importance matrix denoted by *I* as follows:

(25) $$\begin{array}{l} I=\frac{1}{2}\left(\text{a}{\text{b}}^{⊺}+\text{b}{\text{a}}^{⊺}\right). \end{array}$$

**Figure 9:**
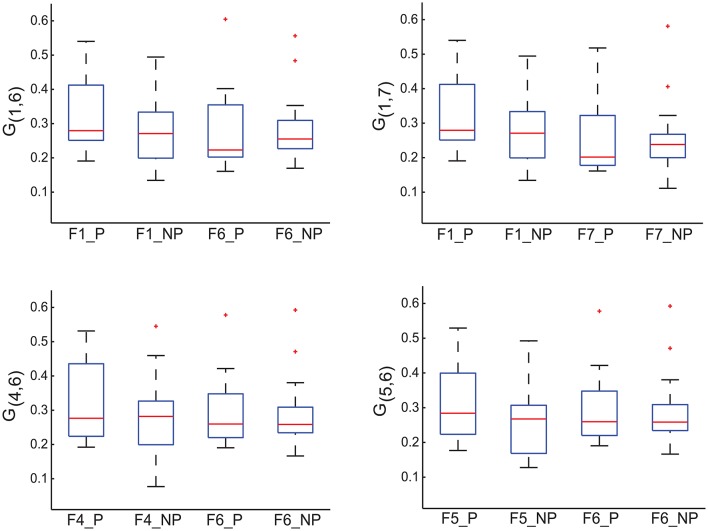
**For the graph matrices generated in thi study, we display four of their matrix elements which are associated with the four edges highlighted in Figure 8**. *G*_1, 6_ in the upper-left panel, *G*_1, 7_ in the upper-right panel, and *G*_4, 5_ in the lower-left panel measure the connectivity of edge *E*_1.6_, *E*_1, 7_ and *E*_4, 5_ (respectively) that form the three-node sub-network. Recall that the task-related importance of this sub-network has significantly increased after training. In contrast, *G*_5, 6_ in the lower-right panel measures the connectivity of edge *E*_5.6_ and its task-related importance has significantly reduced after training. The four boxplots in each panel are associated with pre-training session & patient group, pre-training session & control group, post-training session & patient group, and post-training session & control group (from left to right, numbered as 1, 2, 3, and 4 in the order).

Each off-diagonal element of *I* measures the importance of the corresponding edge in terms of discriminating MCI patients from healthy controls. To identify possible sub-networks that have significantly changed after training, we are first to identify the edges whose importance measure has significantly changed after training. To this end, we generated an ensemble of the selected importance matrices using the procedure that was used to generate an ensemble of the selected GMLVQ metric (tensor) matrices for the relevance feature analysis. Subsequently, we conducted two one-sided sign rank tests for each of the 21 edges to find those edges whose importance values have significantly increased or reduced after training. Denote the edge connecting node *i* and *j* by *E*_*ij*_. This analysis revealed that the importance measure of three following edges has significantly increased: *E*_17_, *E*_16_, and *E*_64_. A significant reduction of its importance measure was observed for *E*_65_. These four edges are displayed in Figure 8. Figure 9 highlighted a subtle difference between the sub network (i.e., *E*_17_, *E*_16_, and *E*_64_) and the single edge *E*_65_. For the three-node sub-network, the connectivity strength is highest for MCI patients before training. For the single edge *E*_65_, the connectivity strength is lowest for healthy controls before training. This suggests that FGF-Frontal-Pre, the most relevant feature in *M*-FGF, could be related to these three-node and single-node sub-networks.

#### 4.3.3. Privileged information

In addition to *M*-CD, *M*-PSC, and *M*-FGF, *M*^+^-CD-PSC, and *M*^+^-CD-FGF were conducted to investigate GMLVQ classification of MCI patients and controls when fMRI features were incorporated as privileged information. The relevance of the four cognitive features in *M*^+^-CD-PSC and *M*^+^-CD-FGF was estimated from the diagonal elements of the metric tensors and displayed in the middle and right panel of Figure 3 (respectively). Though PSC and FGF are two different kinds of fMRI features, we still consistently observed that cognitive inhibition and divided attention are the two most relevant cognitive features. Moreover, the relevance of divided attention is more profound than that of cognitive inhibition. When compared to *M*-CD, cognitive inhibition did emerge as a relevant feature only when the privileged information was incorporated. Also, Figure 4 shows that when compared to *M*-CD, the interplay between divided attention and selective attention became significantly positive in *M*^+^-CD-PSC and *M*^+^-CD-FGF, that is, the experiments in which the privileged information was incorporated.

## 5. Conclusion

In this study, we employed GMLVQ classifiers to discriminate cognitive skills in MCI patients vs. healthy controls using cognitive and/or fMRI data. Specially, we have adopted a “Learning with privileged information (PI)” approach to combine cognitive and fMRI data. In this setting, fMRI data as an addition to cognitive data are only used to train GMLVQ classifier and classification of a new participant is solely based on cognitive data. As the inputs to GMLVQ classifier, the cognitive features include working memory, cognitive inhibition, divided attention and selective attention scores. Also, we extracted three different types of fMRI features from fMRI data as follows: PSC (percent signal change), and SGF (spatially grouped graph feature) and (functionally grouped graph feature).

We first tested our baseline GMLVQ classifier with four cognitive features as inputs. Its classification performance is measured by (25%, 75%) percentile of Macro-averaged Mean Absolute Error (MMAE), that is, (0.32, 0.44). The best of the five fMRI GMLVQ classifiers (i.e., the ones using the fMRI features as their inputs) yields a lower bound of classification error, which is (0.14, 0.31). Interestingly, the best of the PI-guided GMLVQ classifiers (i.e., the ones using the four cognitive features as their inputs and using the fMRI features as privileged information) have achieved (0.23. 0.39). This implies that incorporating fMRI features as privileged information can significantly improve the classification performance of a baseline GMLVQ classifier for classification of cognitive skills in MCI patients vs. controls.

Crucially, we have also performed “relevant feature analysis” for all three GMLVQ classifiers: the baseline GMLVQ classifier, the best fMRI-guided GMLVQ classifier, and the fMRI GMLVQ classifier. For the baseline classifier, “divided attention” is the only relevant cognitive feature for the classification task. When the privileged information is incorporated, divided attention remains the most relevant feature while cognitive inhibition becomes also relevant. The above results suggest that attention-rather than only memory-plays an important role for the classification task. More interestingly, this analysis for the fMRI GMLVQ classifier suggests that (1) among three ROIs used, the frontal ROI is most relevant for the classification task; (2) when the PSC feature as an overall measure of fMRI response to structured stimuli is used as the inputs to the classifier, the post-training session is most relevant; and (3) when the graph feature reflecting underlying spatiotemporal fMRI pattern is used, the pre-training session is most relevant. Further analysis has indicated that training may cause an overall increase of the brain activity only for MCI patients while it may have “mitigated” the difference in brain connectivity pattern between MCI patients and healthy controls. Moreover, these training-dependent changes are most significant for a three-node sub-network in the frontal ROI. Taken together these results suggest that brain connectivity before training and overall fMRI signal after training are both diagnostic of cognitive skills in MCI

Our study employs machine learning algorithms to investigate the neurocognitive factors and their interactions that mediate learning ability in Mild Cognitive Impairment. Our work is not limited to developing and validating machine learning approaches; in contrast it advances our understanding of the neurocognitive mechanisms that mediate learning in health and disease. For example, the role of cognitive inhibition in cognitive profile classification seems to be significantly enhanced when brain imaging information (related to a sequence learning prediction task) is provided as privileged information. This opens questions about the possible interplay between circuits involved in cognitive inhibition and those involved in learning sequence prediction tasks. We also observed significant positive interplay between divided and selective attention when brain imaging data is used as privileged information. No such interplay was detected without the privileged information. Again, this raises interesting questions regarding circuitry involved in sequence prediction and the two attention types.

## Author contributions

Collection of cognitive and fMRI data: ZK, CL. Diagnosis of MCI: PB. Determination of ROIs: CL. Design of the work: PT, ZK, YS. Analysis and interpretation: HA, YS, PT, SF. Drafting the article: HA, YS, PT, SF, ZK, CL. Critical revision of the article: YS, PT, ZK. Final approval of the version to be published: HA, YS, SF, CL, PB, ZK, PT.

### Conflict of interest statement

The reviewer LS declared a shared affiliation, though no other collaboration, with one of the authors ZK to the handling Editor, who ensured that the process nevertheless met the standards of a fair and objective review. The other authors declare that the research was conducted in the absence of any commercial or financial relationships that could be construed as a potential conflict of interest.

## Footnotes

^1^The set of points in the input space is defined by the receptive field of prototype **w**, where this prototype is picked as their winner.
